# Microbiome in heritage: how maternal microbiome transmission impacts next generation health

**DOI:** 10.1186/s40168-025-02186-8

**Published:** 2025-09-26

**Authors:** Clara Delaroque, Benoit Chassaing

**Affiliations:** Microbiome-Host Interactions, Institut Pasteur, Université Paris Cité , INSERM U1306, Paris, France

## Abstract

After birth, the infant’s intestine is colonized by microorganisms, initiating a period of rapid microbial expansion and major compositional maturation influenced by both maternal and environmental factors. Simultaneously, the host’s intestinal environment exhibits unique characteristics that facilitate critical interactions with the developing microbiome during this early-life window. These early biological events have lasting effects on health, fostering immune tolerance to environmental exposures or, conversely, increasing susceptibility to noncommunicable diseases—such as allergies, obesity, and inflammatory bowel disease—if microbiome development is disrupted. In this review, we summarize recent advances in understanding the key stages of microbiome development after birth and explore how changes in the maternal environment—especially diet—as well as maternal intestinal bacteria and their derived molecules shape the microbiome’s composition and function in early-life, ultimately influencing long-term health and disease risk.

Video Abstract

**Supplementary Information:**

The online version contains supplementary material available at 10.1186/s40168-025-02186-8.

## Introduction

Modernization and industrialization have profoundly transformed human lifestyles, impacting diet, hygiene practices, and medical care. While these advancements have significantly improved the prevention and treatment of infectious diseases, they have been paralleled by a marked increase in the incidence of non-communicable diseases (NCDs) [1–3]. Notably, autoimmune conditions such as type 1 diabetes and multiple sclerosis, as well as diseases with a chronic inflammatory component including obesity, type 2 diabetes, and inflammatory bowel disease (IBD), have risen dramatically over the past few decades [4–10]. Emerging evidences suggest that disruptions in the early-life interactions between the gut microbiome and the host’s intestinal environment may contribute to the development of these diseases, thus potentially contributing to the rising rates of NCDs [11, 12]. These interactions involve not only the microbiota, describing the intestinal microbial ecosystem, but also relies on its specific activities and derived compounds as well as its response to surrounding environmental factors; altogether designated as the microbiome which will be the focus of this manuscript [13].

As human are born, they are inoculated with microorganisms, among them some being from maternal origin. This community will colonize all body sites and develop extensively during the first years of life, undergoing major gradual changes in composition and richness [14]. The maternal microbiome’s contribution to the one of her offspring is reflected in the significant proportion of microbial strains shared between mothers and their children, a pattern not observed when comparing unrelated individuals within the same household [15]. Moreover, during early-life development, the mother plays a crucial role in shaping the offspring's microbiome through shared environments and breast milk components. This maternal influence on the next generation’s microbiome is gaining increasing attention, as a well-balanced microbiome during the early life period is essential for proper interactions with the developing mucosal immune system. These interactions are critical for establishing immune tolerance, and disruptions in this process have been linked to long-term consequences, including increased susceptibility to a broad range of NCDs [16–19]. Consistent with this concept, growing evidence underscores the influence of maternal factors, such as diet, on the modulation of the offspring’s developing microbiome [20–24]. In this review, we first explored the parallel development of the gut microbiome and the host’s immune system during the early-life period, highlighting their unique characteristics compared to adults. We then detailed mechanisms through which maternal factors are influencing the neonatal microbiome, emphasizing the role of maternal microbiome in shaping the early-life microbiome. Finally, we discussed the long-term consequences of early-life perturbations of host-microbiome interactions, specifically those induced by maternal factors, and outline current knowledge gaps and potential avenues for therapeutic intervention during this critical developmental window.

## The early-life microbiome and immune compartment develop concomitantly and interdependently

### Establishment of the intestinal microbiota

Under normal conditions, the fetal gastrointestinal tract is suspected to be sterile, with the first exposure of the host mucosal surfaces to commensals occurring during the birth process [25]. The maternal microbiota, from various origins (vaginal, skin, intestinal, etc.) hence constitutes the first microbial inoculum, and from birth, first colonizers include facultative anaerobes bacteria such as *Escherichia coli* and *Bifidobacterium* that create a new environment to promote the colonization of strict anaerobes [26–29]. This is next followed by colonization with *Bacteroides*, *Clostridium*, and *Bifidobacterium* spp. The intestinal microbiota of neonates is characterized by low diversity dominated by the phyla *Proteobacteria* and *Actinobacteria*, with specific metabolism enabling simple carbohydrate degradation and amino acid transportation [27]. In the subsequent months, as solid food is introduced, microbiota diversity increases gradually, with the emergence and expansion of *Firmicutes* and *Bacteroidetes*, as well as appearance of species known for their capacity to ferment different carbohydrates including *Faecalibacterium prausnitzii* [27]. These changes induce a metabolic shift toward complex carbohydrate degradation, short-chain fatty acid (SCFAs) production, and amino acid biosynthesis [14, 29–32]. By the end of the first year of life, infants possess an individually distinct microbial profile, converging toward the characteristic of an adult microbiota, reaching complete stability by 2–5 years of age [14, 32], even if such stable status is a relative concept. A more precise description of microbiota development from birth to an adult-like state has been hindered by the significant interindividual variability observed in the human population, which results in this establishment not being universal at the strain level. Moreover, microbiota analysis techniques—including 16S rRNA amplicon sequencing, metagenomics, and culture-based approaches—as well as heterogeneity in DNA extraction methods, sample collection, and storage conditions, have all been reported to introduce variability in microbial composition [33–35]. These factors must therefore be carefully considered when interpreting results or comparing data across studies. However, despite population diversity and pre-analytics/analytics-induced variation, key clusters have been identified as keystone steps in microbiota development in children [31]. Such approach, undertaken in the study by Stewart et al. identified in The Environmental Determinants of Diabetes in the Young (TEDDY) study 10 microbiota clusters, which were strongly associated with children’s age, leading to the identification of three distinct phases of microbiome progression: a developmental phase (months 3–14), a transitional phase (months 15–30), and a stable phase (≥ 31 months) [31]. In terms of composition, all five phyla change significantly in the developmental phase, revealing the high dynamism and microbiota remodulation ongoing during this phase. During the transitional phase, only *Proteobacteria* and *Bacteroidetes* changed significantly, while in the stable phase, composition were observed to be relatively stable, suggesting that stabilization is reached by the human adult-like microbiota after this early-life period [31].

Early-life microbiota development is shaped by a myriad of factors, with initial colonization playing a pivotal role. Compared to vaginally born infants, those delivered by C-section exhibit significant compositional differences, with an enrichment in skin-associated bacteria [14, 29, 36–38]. As the microbiota develops, the impact of the delivery mode diminishes [14, 29, 36, 38], while the high dynamic nature of the early-life microbiota during its establishment makes it particularly susceptible to environmental influences, including maternal factors. Such critical window of microbial development coincides with the maturation of the host’s intestinal compartment, especially the mucosal immune system, which depends on proper interactions with the evolving gut microbiota [17, 18, 39].

### The early-life intestinal compartment is shaped to foster microbiome development

The intestinal epithelium and mucosal immune system of newborns are drastically different from the one of adults, resulting in responses to similar stimuli that can drastically vary according to the developmental stage of the individual. This is for example highlighted by the increased susceptibility of newborns to infections compared to adults [40].

**Fig. 1:**
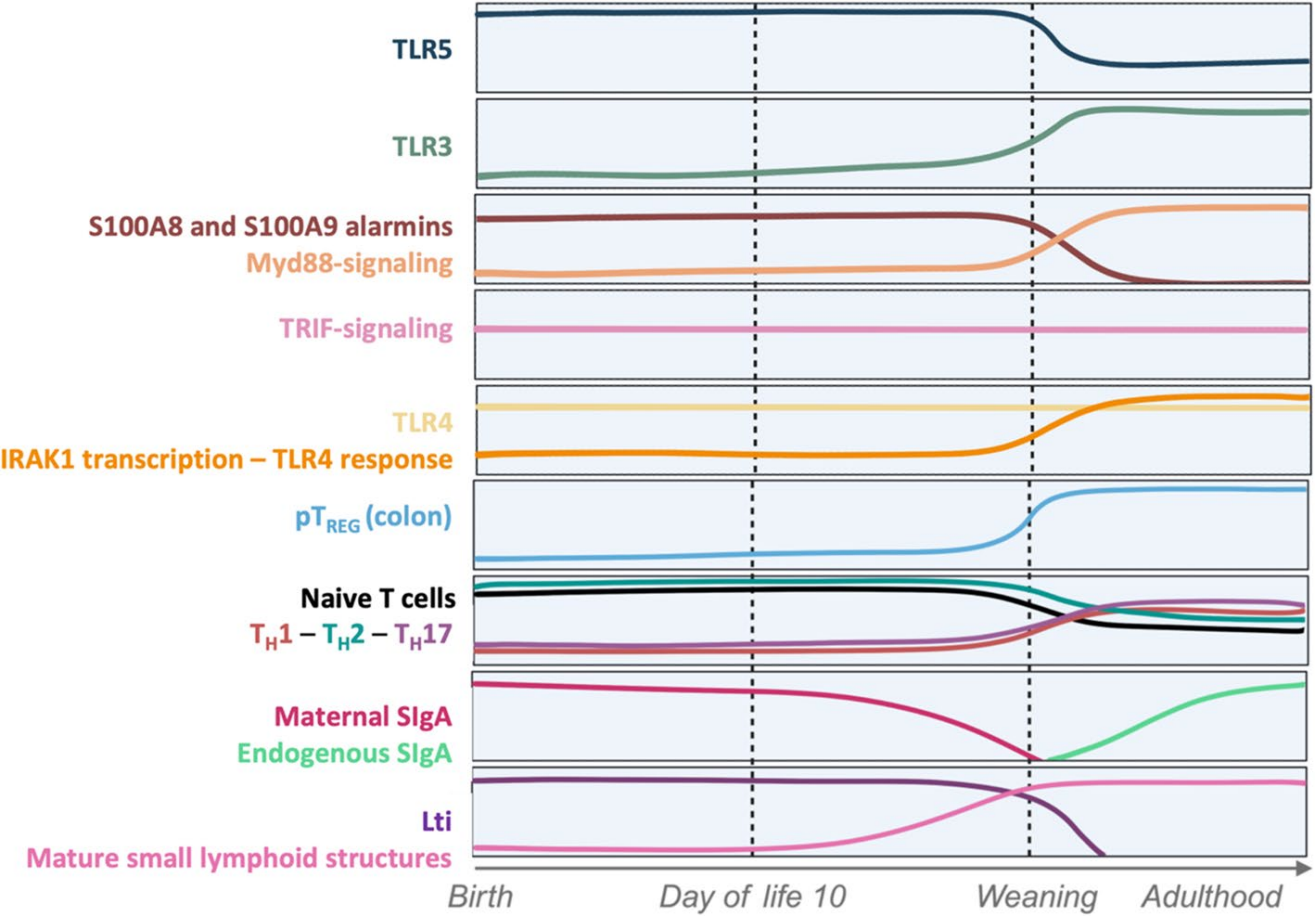
Early-life microbiota sensing machinery and associated intestinal immune reaction. The early life period is characterized by distinct molecular and cellular processes, defining a unique developmental window. Before weaning, TLRs levels and downstream signaling pathways differ significantly from those in adults. These differences help to regulate inflammatory responses to newly encountered microbes while promoting immune tolerance. In addition to variations in microbiota and intestinal compartments, composition and function of the intestinal immune system are uniquely adapted during early life. T cell populations, along with the balance between immune precursor structures and mature immune components, undergo significant changes around weaning, marking the transition toward an adult-like immune state. Such shift is also accompanied by the initiation of endogenous secretory IgA production, as maternal milk-derived SIgA are no longer delivered. TLR, toll-like receptor; MyD88, myeloid differentiation primary response 88; TRIF, TIR-domain-containing adapter-inducing interferon-β; IRAK, interleukin-1 receptorassociated kinase; SIgA, secretory immunoglobulin A; LTi, lymphoid tissue inducer

The first notable difference in the early life period is the differential expression of many pathogen recognition receptors (PRRs) compared to the adult intestine (Fig. 1) [41–45]. Indeed, the expression of several toll-like receptor (TLR)-encoding genes by the intestinal epithelium undergoes significant changes with age. For instance, Fulde et al. reported that the gene encoding the flagellin-specific receptor TLR5 is expressed 100 times more during early life [44, 46], whereas Tlr3 expression increases gradually after birth [42]. When comparing the expression profiles of genes encoding TLRs in the mouse intestine between conventional and germ-free mice, differences were observed as Tlr1, Tlr4, and Tlr9 were found to be decreased in germ-free animals, suggesting microbiome-dependent regulation of such genes expression [42]. Additionally, the signaling pathways and subsequent immune responses triggered by TLR binding by their ligands are highly specific to the early-life period. High amounts of the perinatal alarmins S100A8 and S100A9 have been reported to specifically alter MyD88-dependent proinflammatory programs, thus preventing hyperinflammatory responses (Fig. 1) [47]. However, such modulation of the immune response to microorganisms does not affect TIR domain containing adaptor inducing interferon-β (TRIF)-mediated pathways, resulting in a selective, transient microbial unresponsiveness that prevents harmful hyperinflammation in the delicate neonate while allowing for sufficient immunological protection [47]. Such tight regulation of TLR signaling has also been described for TLR4 in early-life, as epithelial TLR4 signaling is reprogrammed following the production of the small mRNA miR-146a prior to weaning (Fig. 1) [48]. This reprogramming inhibits the transcription of the TLR signaling molecule IL-1 receptor-associated kinase (IRAK) 1, thus dampening the TLR signaling cascade that results in pro-inflammatory genes expression [48].

In addition to microbiome sensing by the host in early life, the response elicited by the neonatal immune system also drastically differs from adults. The early-life period has indeed been reported to be a specific window for the induction of tolerogenic regulatory T cells (T_REG_) directed toward microbiota members. Neonatal CD4^+^ T cells are more prone than adult CD4^+^ T cells to differentiate into T_REG_ cells upon stimulation by luminal antigen in the colon [49, 50], making the early-life period a specific and crucial time for tolerance induction towards commensals. This phenomenon has also been observed in humans, as the effectiveness of peanut sensitization trials—designed to induce a tolerogenic response to peanut allergens and reduce the development of peanut allergies—has been shown to be highly dependent on the child’s age at the time of intervention [51, 52]. The greatest reduction in allergy development was observed in younger children, with the protective effect diminishing as the intervention was conducted at older ages [52]. This trend suggests that older children may have a reduced capacity to develop long-term tolerance to the exposed antigens [52]. The early-life mucosal T cell compartment is biased toward T_H_2 response, which is promoted by natural killer T (NKT) cells [53], with IFNɣ-producing T_H_1 and IL-17-producing T_H_17 cells, only observed after weaning (Fig. 1) [54]. Such T_H_2-bias is inhibited by the intestinal microbiome, with the T_H_1 to T_H_2 balance being reversed toward adult-like balance upon weaning as the microbiome becomes more complex (Fig. 1). Prior to weaning, a majority of T cells in the early-life intestine remains immature throughout the postnatal period, which is highly dependent on both T_REG_ and maternal secretory immunoglobulin A (SIgA) [55, 56]. Such suppressive mechanisms to prevent inflammation during the early-life period also relies on the high abundance of erythroid CD71^+^ cells, which suppress myeloid and lymphoid cell activation in the intestine following bacterial colonization [57]. Humoral immunity specificities during the early-life period also contribute to the promotion of microbiome development. While B cells populate the intestine during the postnatal phase, functional IgA-producing plasma cells only emerge after weaning, after the first year of life in humans (Fig. 1) [58–60]. This delayed maturation of antibody-secreting B cells is both compensated for and regulated by luminal SIgA derived from maternal milk [58, 60], which play an active role in microbiome development. Hence, the early-life mucosal immune system is strongly inclined toward tolerance and hypo-responsiveness, minimizing inflammation in response to initial colonization in order to foster the development of a stable microbiome.

### Host-microbiome interactions during the early-life period: the central role played by colonic goblet cells

**Fig. 2:**
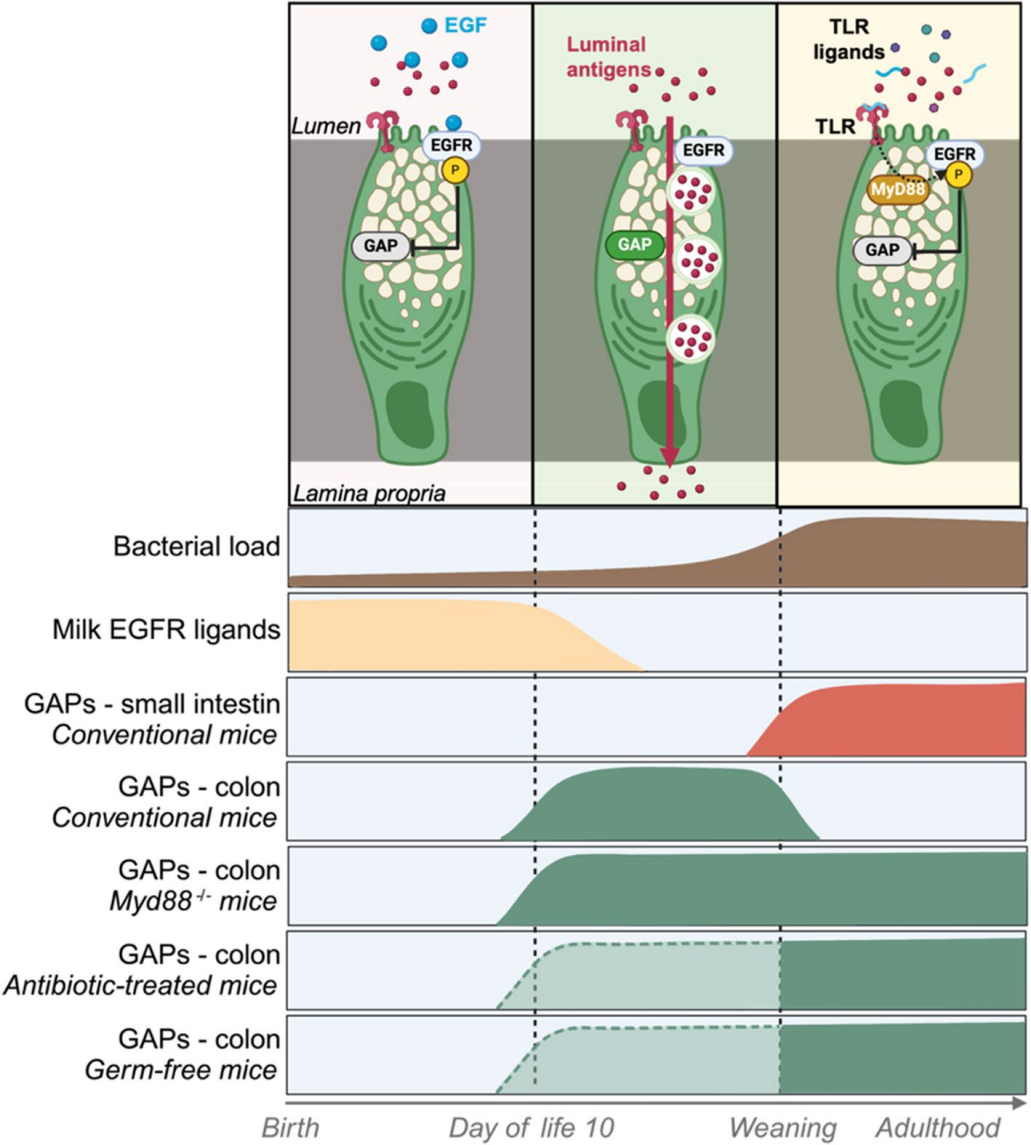
Goblet-cell-mediated regulation of host-microbiota communication during the early-life period. Goblet cell associated antigen passages (GAPs) are tightly regulated by maternal milk-derived EGFR ligands and microbial signals via the MyD88 adaptor protein. This regulation defines three distinct phases for luminal antigens—originating from microbiota or diet—to traffic from the lumen to the lamina propria, where they are encountered by intestinal immune cells. As a result, in mice, GAPs are present in the colon only between day 10 and weaning, which coincides with early-life propensity for regulatory T cell induction, promoting long-lasting tolerance to microbial and dietary antigens. In MyD88-deficient mice or those with microbiota depletion due to antibiotic treatment or germ-free conditions, GAP levels are impaired after weaning. Areas framed by dashed lines represent hypothetical deductions that require further investigation. GAP, goblet cell–-associated antigen passage; EGFR, epidermal growth factor receptor; MyD88, myeloid differentiation primary response 88

The neonatal delicate balance between commensals sensing while preventing dissemination of potentially harmful bacteria is further supported through strict control of mucosal immune cells exposure to luminal antigens. Timely regulated mechanisms for antigen translocations through goblet cells in the colon have resulted in the identification of different antigen-exposure phases, which frame the early-life window of opportunity for tolerance (Fig. 2). As described in an elegant study by Knoop et al. such phases correspond in mice to:

i. No luminal antigen encountered in the small intestine nor colon between birth and 10 days of life.
ii. Antigens encountered exclusively by the colonic immune system until weaning.
iii. Antigens encountered exclusively in the small intestine postweaning (Fig. 2) [39].

Such antigen availability appears to be mediated by its trafficking through goblet cell associated antigen passages (GAPs), which are tightly timely regulated [39, 61, 62]. GAPs are formed through acetylcholine (ACh)-dependent endocytic events, facilitating the transcytotic delivery of luminal fluid-phase components across the cell [61–65]. In goblet cells, ACh signaling is inhibited by the activation of the epidermal growth factor receptor (EGFR). In the intestine, such inhibition can occur either through the intrinsic sensing of gut microbial products via goblet cells TLRs or by the presence of EGFR ligands in the lumen [61, 62]. Consequently, during the early life period, GAP formation is suppressed for the first 10 days due to EGFR activation by ligands derived from maternal milk (Fig. 2) [39, 63, 66]. As the concentration of EGF ligands in maternal milk declines, ACh signaling becomes sufficient to induce GAP formation in the colon but not in the small intestine (Fig. 2). This allows for selective luminal antigen translocation between day 10 and weaning. Upon weaning, the introduction of solid food leads to a surge in bacterial load, triggering TLR stimulation in goblet cells and subsequent Myd88-dependent EGFR activation in the colon (Fig. 2) [39, 63, 64]. This marks the end of luminal antigen trafficking through goblet cells in the colon. However, in the SI, GAPs open due to the cessation of maternal milk intake, facilitating antigen translocation in this region (Fig. 2) [39]. Disruption of Myd88 signaling—whether through *Myd88* depletion (*Myd88*^*−/−*^ mice), antibiotic treatment, or germ-free status—has been reported to result in the persistence of colonic GAPs into adulthood (Fig. 2) [39, 61, 64, 65], highlighting the importance of precise regulation of host-microbiome interaction through this route during the early-life period.

## Maternal modulation of microbiome development

During pregnancy, the mother significantly influences the developing fetus in a way that ultimately impacts microbiome development after birth. Indeed, *in utero* immune priming has been observed in offspring from transiently-colonized dams compared to offspring originating from germ free dams [67, 68]. This was associated with the capacity of maternal microbiome to influence the fetus during pregnancy though IgG-bound microbiome-derived antigen crossing the placental barrier to prime fetal immune cells [67], as well as SCFA influencing cell differentiation through binding of SCFA receptors on embryonic cells [68]. Such immune priming results in changes of expression in genes involved in antimicrobial peptides activity, mucus secretion, and innate immune response, all known to impact intestinal microbiome development. In addition to *in utero* influences, the nursing period also represents a critical window during which maternal factors are shaping the offspring’s microbiome. Although prenatal maternal perturbations—such as stress, diet, or antibiotic treatment—have been reported to affect the microbiome and immune development of the offsprings, comparative studies involving pre- and postnatal antibiotic exposure, as well as cross-fostering experiments (where newborn mice are transferred to a different nursing dam at birth), indicated that the offspring’s microbiome more closely resembles that of the nursing dam rather than their biological mother [22, 69–71]. Moreover, disruptions in the nursing dam’s microbiome, rather than that of the birth mother, have been associated with altered microbiome development and impaired immune development in the offspring [22]. These findings importantly suggest that maternal influence during the nursing period may outweigh *in utero*/perinatal factors in shaping long-term health outcomes [70, 72], also both contributing to microbiome and health of the next generation, as previously reviewed [73].

### Direct strain transfer as maternal contribution to offspring’s microbiota

**Fig. 3:**
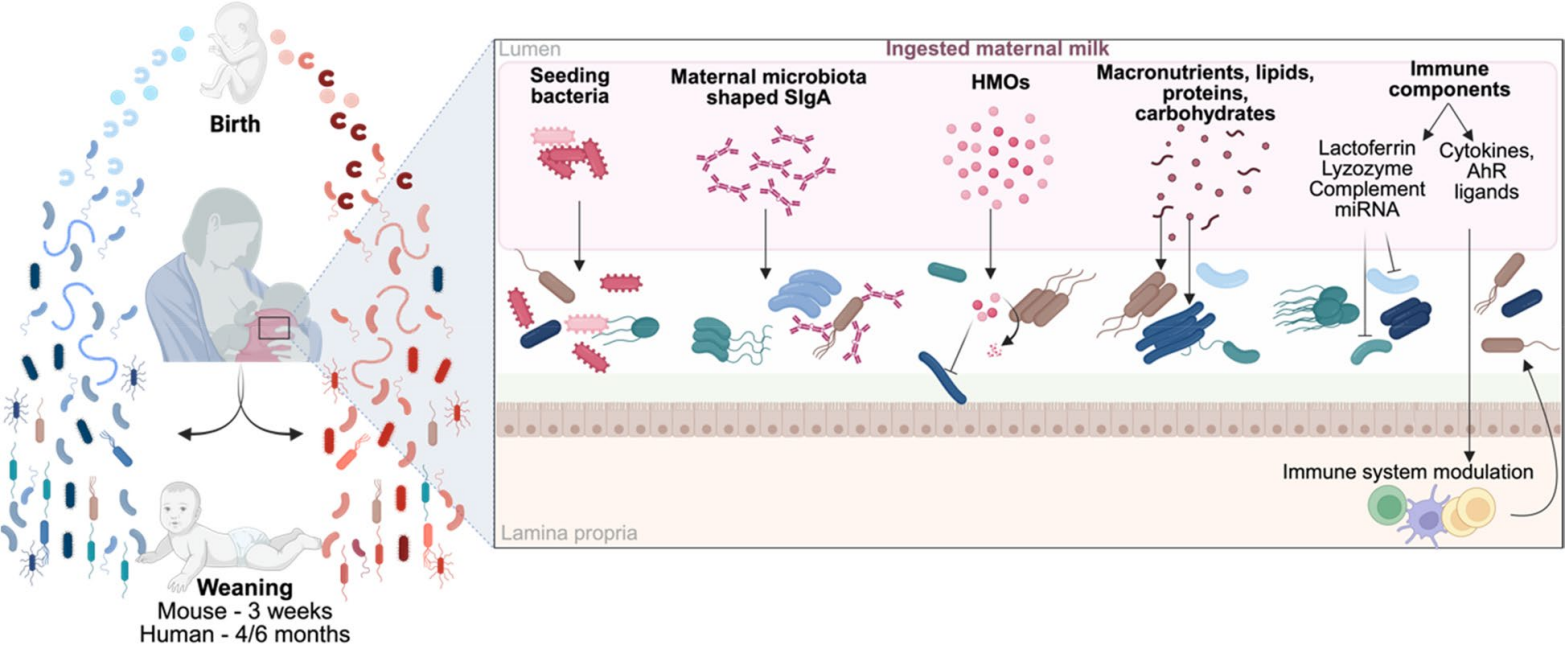
Impact of maternal milk components on microbiota establishment. Maternal milk plays a crucial role in the development of the offspring's microbiota, providing a range of components that influence bacterial growth and colonization. It contributes to the infant’s microbiota by supplying bacteria, SIgA, HMOs, nutrients, and immune factors, which together promote or inhibit the expansion of specific bacterial populations through both direct and indirect mechanisms. The interplay of these factors shapes the overall development of the microbiota in the offspring. SIgA, secretory immunoglobulin A; HMOs, human milk oligosaccharides; miRNA, microRNA; AhR, aryl hydrocarbon receptor

As the mother constitutes the first source of bacterial strains for offsprings in mammals, many studies have reported the significant similarities between maternal and offspring microbiota composition. It was for example previously reported that strains are shared between a mother and her child [15, 74–77], with the transmission rate of strains, calculated as the amount of strains found in both mother and child, being higher between them than with unrelated mother, father, or individuals living in the same household in early-life [15, 74, 78], but not later, suggesting microbiota transfer between both parents and children after early infancy [77]. Such transgenerational microbiota transmission appears to be more effective in early life and diminishes as children grow and develop their own, personalized and adult-like, microbiota [79]. This suggests that maternal influence is particularly significant during the early stages of microbiota development. Hence, strains transmission, in addition from shared genetic and similar environment, results in infant’s fecal microbiota composition to be significantly more similar to that of its own mother than to that of other mothers over the entire first year of life [38], which may in part result from maternal strains persistence during the first year of life [77]. However, it is important to note that such strain transmission efficiency is highly impacted by birth mode, with maternal contribution to infant’s microbiota in C-section delivered-babies being significantly lower [38, 76], promoting the colonization of non-gut bacteria [77], which highlight the important role of delivery-associated strain transmission in maternal contribution to offspring’s microbiota. Complementary to this initial colonization, maternal breast milk microbiota also contributes to strain transfer across generations. Indeed, through daily consumption of approximately 800 mL of maternal milk, infants ingest between 10^5^ and 10^7^ bacteria per day, representing a non-negligeable input of bacterial strains (Fig. 3) [80]. The human breast milk comprises nine predominant genera—*Staphylococcus*, *Streptococcus*, *Serratia*, *Pseudomonas, Corynebacterium*, *Ralstonia*, *Propionibacterium*, *Sphingomonas*, and *Bradyrhizobium*—which collectively account for approximately half of the microbial community in milk [80]. However, their relative abundance varies across individuals and lactation period [81–84]. It is also important to note that the low microbial biomass in breast milk drive significant challenges for the study of milk-associated bacteria, since many of the identified bacterial taxa are often indistinguishable from environmental and skin contaminants [85]. In a study investing milk microbiota contribution to infants microbiota in a cohort of 34 mother–infant dyads, Laursen et al. reported that 1/3 and 1/5 of the bacterial taxa detected in infant’s feces were shared with the corresponding mother’s milk at 5 and 9 months of age, respectively, with *Streptococcus*, *Veillonella*, and *Bifidobacterium* spp. among the most frequently shared [83]. Of note, these findings have to be interpreted carefully as bacteria identification was assessed leveraging 16S rRNA gene sequencing, preventing formal identification of shared strains between milk and the infant’s microbiota.

### Human milk oligosaccharides and microbiome development

In addition to providing a source for bacterial strains, maternal milk also contains an array of nutritive and immune components that have been reported to directly influence microbiome development of the next generation. Among these components are human milk oligosaccharides (HMOs), which constitute the third largest nutrient fraction of breast milk, comprising 5–20 g/L in mature milk [83]. HMOs can reach the intestinal tract undigested, becoming available as substrates for the growth of specific gut microbes (Fig. 3). Studies investigating correlations between the HMO profile of ingested milk and microbiome composition in children have observed that several of the most abundant genera detected at 5 months of age correlate with the relative abundances of select HMOs [83, 86]. This suggests that HMOs can directly impact microbiome composition by favoring bacterial strains capable of degrading and using HMOs as an energy source. In addition, HMO can function as soluble receptors [87] or modify cell-surface glycans [88, 89], thereby modulating the attachment of specific bacteria to intestinal epithelial cells in a strain-dependent manner [89–93]. HMO’s impact on infants’ microbiome composition and function can be illustrated with the example of *Bifidobacterium longum*, which is consistently present in infants'microbiome. However, strain-level analysis has revealed consistent subspecies replacement patterns associated with lifestyle and age, but also breastfeeding practices, and which are characterized by changes in HMO-degrading capabilities [14, 29, 83, 94–97]. Variation in the presence of genes encoding enzymes required for HMO degradation versus dietary substrates in the genomes of *B*. *longum* subspecies provide it with different advantages in colonizing the gut of breastfed vs formula-fed children, as well as throughout the early-life period and the introduction of solid food at weaning [94–96]. As an example, subspecies of *Bifidobacterium longum*, such as *B*. *longum* subspecies *longum* or *infantis*, differ in their genomic capacity to use HMO or food-derived substrates, leading to *B*. *longum* subspecies *infantis* being associated with breastfed children [14, 29, 83, 94, 95, 98]. Based on genetic investigations, *B*. *longum* subsp. *infantis* is particularly adapted to use HMOs, while *B*. *longum* subsp. *longum* and *breve* tends to colonize later in life, as breast milk intake decreases and solid foods are introduced [14, 29, 83, 94, 95]. Additionally, a transitional *B*. *longum* subspecies has been identified, which harbor the genetic material allowing the use of both HMOs and food-derived substrates, leading to its expansion during the weaning period [94]. Notably, this transitional subspecies, as well as other *B*. *breve* subspecies, have been predominantly observed in samples from non-industrialized countries, underscoring the influence of environmental and lifestyle factors—beyond maternal determinants—on microbiome development [96, 97].

### Milk-derived secretory IgA

Mother’s milk SIgA delivered to infants during breastfeeding are crucial in shaping and modulating immature infants’ microbiome (Fig. 3). The highest concentration of SIgA is reported in milk produced shortly after birth (days 2–5, with approximately 2.5 g/L), and subsequently slightly decline through time to reach approximately 0.7 g/L in mature milk around 30 days after birth [99]. In preparation for lactation, IgA-secreting plasma cells are recruited to the mammary gland. Gut plasma cells have a higher propensity to migrate toward mammary glands, thus providing the milk with IgA specific for antigen found in the maternal microbiome [56, 100–102]. Epithelial cells within the mammary glands express pIgR, enabling secretion of the full SIgA molecule directly into the milk [103]. As a result, the IgA repertoire of milk SIgA and maternal intestinal SIgA share more similarities than repertoires from other body sites. Upon weaning, plasma cells accumulation declines rapidly in the mammary gland. Hence, through milk ingestion, the infant's microbiome is exposed to a wide array of SIgA, which modulates the expansion and function of specific microbiota members, with various mechanisms at play. Briefly, SIgA can induce bacterial agglutination or neutralization, can provide an additional carbon source, and can facilitate bacterial adherence, altogether exerting a major shaping force on the developing microbiome [104, 105]. It is important to note that human milk is a major source of SIgA in early life, as infants have very low levels of their own IgA at birth and it only gradually rises in the first few months of life when the immune system develops [56, 60, 106, 107]. This is further evidenced by the significantly decreased proportion of fecal IgA in formula-fed young children compared to age-matched breastfed infants [108]. Considering the ability of SIgA to modulate bacterial strains colonization, absence of maternal SIgA in the gut of formula-fed children is suspected to contribute to the observed microbiome differences between these children.

### Other milk-derived compounds influencing the infant’s microbiome development

Many other milk-derived components have been suggested to have an impact on the developing microbiome. This is the case for macronutrients, including lipids, proteins and carbohydrates (Fig. 3). While most of these components are absorbed in the small intestine, sparse evidences suggest that they could nonetheless influence microbiota composition through inhibitory activities, or serve as selective substrates for the growth of specific bacteria [109–112]. Moreover, maternal milk comprises many immune factors in addition to SIgA that were associated with changes in the infant’s microbiome (Fig. 3). This comprise lactoferrin, that has a direct cytotoxic effect against a large panel of microorganisms [113], lysozyme able to lyse gram positive bacteria [114, 115], as well as cytokines and aryl hydrocarbon receptor (AhR) ligands influencing the infant’s immune system, thus indirectly impacting the microbiome [116–118]. In addition, breast milk also contained immune complement components that were shown to directly lyse specific members of gram-positive gut commensal microbiota [119]. More recently, significant associations have been observed between milk miRNA and infants microbiota members abundance, suggesting that milk-derived miRNA may also contribute to modulate offsprings’ microbiota development [120]. While these correlations do not establish a causal relationship between milk-derived miRNAs and the offspring’s microbiota, recent studies have reported gene expression modulation in both cell lines and bacteria when milk-derived miRNAs were supplemented to the culture media [121, 122]. These findings interestingly suggest a potential direct effect of milk-derived miRNAs on both the host and its microbiota, although the underlying mechanism(s) will require further investigation. It is important to note that maternal milk composition is highly individual, as it is influenced by numerous factors including diet [123–125]. Additionally, milk composition changes as time progresses after birth, altogether further increasing the complexity of how milk modulates the microbiota of the next generation.

### Maternal environment shapes the offspring’s microbiome development

As a result of the complex maternal regulation of an infant’s microbiome—through mechanisms such as bacterial seeding and milk component impact on offspring intestinal bacterial communities—recent evidences highlighted the impact of maternal environmental factors and health on the offspring’s microbiome. This is the case for associations between maternal health status and microbiome in the offspring. Indeed, association have been found between maternal body mass index [124, 126, 127] or gestational diabetes [72, 128, 129] with microbiota specific composition and development patterns both in mice and human studies. Moreover, maternal exposure to antibiotic treatment has also been described as having a significant impact on offspring microbiota development, with microbiota of offspring from antibiotic exposed mother harboring significant differences in composition and diversity [130–132].

More recently, accumulating evidence points to a significant impact played by maternal diet on offspring’s microbiome, as summarized in Table 1. Dietary fiber intake, widely recognized in the literature for its strong influence on microbiome composition and metabolic activity, has been reported to shape distinct microbiome signatures in offspring from mothers exposed to fiber deprivation. A study by Zou et al. reported, in mice, that offspring born to mothers consuming a low-fiber low-fat diet or a low-fiber high-fat diet during lactation exhibited microbiota composition distinct from those of offspring from dams fed a control diet. This effect was primarily driven by fiber deprivation rather than the fat content of the diet [23]. Of note, the impact of maternal dietary fiber intake on infant microbiome and subsequent health in the human population is currently being investigated within the FeFiFo-MOMS study, which involves 135 healthy women recruited in early-pregnancy up to 18 month after birth, who are randomly assigned to four diet arms [133]. Given the previously discussed importance of host-microbiome interactions during early life, the microbiome’s ability to stimulate host innate receptors appears as a crucial aspect to consider when evaluating early-life microbiota development. In a recent study, we for example reported that maternal consumption of dietary emulsifiers—food additives previously shown to disrupt the microbiome in a way that enhances its pro-inflammatory potential [134–137]—in mice is sufficient to lead to increased microbiota-derived flagellin, as well as bacterial encroachment into the mucus layer in the offsprings at weaning, alongside with compositional changes. This suggests that microbiota function, particularly its ability to stimulate the host immune system, may be strongly influenced by maternal diet [22]. Additionally, maternal diet has been shown to alter maternal milk composition, as reviewed by others [82, 125], suggesting that the impact of maternal diet on offspring microbiome likely arises from a combination of multiple factors.

**Table 1:** Maternal diet on offspring’s microbiome

Maternal diet	Species	Study design	Impact on offspring’s early-life microbiome	Reference
Fiber deprivation	Mouse (C57Bl/6)	Lactating dams fed low-fiber diet	Reduced ɑ-diversity; increased abundance of Proteobacteria; increased bacterial penetration in inner colonic layer	[23]
	Mouse (C57Bl/6)	Dams fed low-fiber diet during gestation and nursing period	Shift in β-diversity, lower fecal SCFA levels	[166]
	Mouse (Swiss Webster)	Dams colonized with minimal microbiota fed fiber-free diet	Delayed colonization with mucin-degrading *Akkermansia muciniphila*	[24]
Calories restriction	Mouse (C57Bl/6)	30% maternal calorie restriction during the second half of gestation	Shift in β-diversity, decreased relative abundance of *Akkermansia* and *Sutterella*; increase in *Anaerostipes* and *Paraprevotella*	[198]
Grain-based diet	Mouse (C57Bl/6)	Maternal grain-based vs purified diet during lactation	Shift in β-diversity, higher relative abundance of *Bacteroides* and lower *Faecalibaculum*	[199]
High-fat diet	Human	Mothers whose intake of fat significantly differed from the mean were separated into a control group (n =13) and a high-fat group (n = 13)	Maternal high-fat diet associated with shift in β-diversity; enrichment of *Enterococcus; *reduction of *Bacteroides*	[200]
	Mouse (C57Bl/6)	Dams fed high-fat diet	Increase in *Firmicutes* and *Betaproteobacteria*; decrease in *Gammaproteobacteria*	[170]
	Mouse (C57Bl/6)	Dams fed high-fat diet	Shift in β-diversity, reduced richness	[173, 174]
Gluten-free diet	Mouse (NOD)	Dams fed gluten-free diet during gestation	Shift in β-diversity, enrichment in *Akkermansia muciniphila*	[201]
Dietary emulsifiers	Mouse (C57Bl6)	Dams exposed to dietary emulsifiers (CMC, P80) during pregnancy and nursing period	Shift in β-diversity, increase fecal flagellin load; increased bacterial penetration in inner colonic layer	[22]
Non-nutritive sweeteners	Mouse (C57Bl6)	Dams exposed to non-nutritive sweeteners (sucralose, acesulfame-K) during pregnancy and nursing period	Significant changes in β- and ɑ-diversity; increase in firmicutes and decrease in *Akkermansia* muciniphila; changes in fecal microbiota-derived metabolites	[177]

## Early-life microbiome perturbation results in lasting health consequences

### Early-life microbiome perturbations promote non-reversible disease susceptibility in adulthood

Impairment in immunomodulatory microbial stimulation of host intestinal compartment has been reported to contribute to irreversible lifelong enhanced disease susceptibility [11, 138]. This was first suggested with the observation that while most abnormalities in germ-free animals can be reversed by intestinal colonization at any age, the ability to restore certain cellular defects that occur in the absence of microbiome is restricted to a short interval in early life, suggesting the existence of a window of opportunity [139]. Indeed, alterations of the early-life microbiome through antibiotics or germ-free conditions have been reported to increase susceptibility to a wide array of diseases, including colitis [17, 140–142], asthma and allergies [17, 143–145], autoimmune diseases such as psoriasis and type 1 diabetes [146, 147], as well as metabolic alterations [16, 148, 149]. Notably, deleterious health effects of early-life microbiome alterations appear to be highly sex-specific, with males and females exhibiting different long-term susceptibilities to diseases such as obesity, immune dysregulation, and neurological impairments [16, 150, 151]. However, colonization or restoration of a proper microbiome after the weaning period has been reported to not being sufficient to prevent such increased disease susceptibility in adulthood [16, 17, 53, 140]. This importantly suggests that long-term health depends on proper host-microbiome interactions during the early-life period. The lasting consequence of early-life microbiome perturbation involves multiple mechanisms, with T cell tolerization in early life playing a central role in sustaining long-term protection against disease development. For example, while colonizing germ-free mice during the critical window of opportunity was sufficient to prevent colitis susceptibility, microbiome restoration combined with T_REG_ depletion failed to confer the same protection [17]. This suggests that early-life microbiome-induced T_REG_ are crucial for maintaining health later in life [17, 39, 152]. Such microbiome-specific T_REG_ induction have been described as a part of the first vigorous reaction of the host to its microbiome, characterized by a pick expression of *Tnfa* and *Ifng*, that is programmed to occur around weaning and is necessary for the normal development of the immune system [17]. In another study, artificial GAP closure in early life was reported to prevent the expansion of pT_REG_ directed toward microbiota members, which was associated with lasting susceptibility to colitis [39]. Such pT_REG_ induction could not be rescued upon colonic GAP opening in the post-weaning period [39], suggesting that it requires both microbiome-derived signals and the specific window of opportunity to occur.

Based on array of evidence originating from mice studies pointing at the potent impact of early-life microbiome perturbation on health, significant research efforts have focused on evaluating whether such early-life perturbated exposure to microbes were associated with susceptibility to disease later in life in human. First, several studies have highlighted the associated exposition to farm environment in childhood and the decreased susceptibility to IBD and allergic risk [153–155], also these studies did not control for genetic nor family history of disease. Moreover, antibiotics treatment during the first year of life was associated with increased susceptibility to a wide array of diseases, including asthma, eczema and allergies [156, 157], as well as IBD [20, 158–160] and metabolic diseases such as obesity and type 2 diabetes [161, 162]. While having a more modest effect on the early-life microbiome compared to antibiotic treatment, C-section delivery was also associated with obesity, type 1 diabetes, Celiac disease, and allergy risk later in life [163–165]. However, the increased inter-individual heterogeneity that exist in the human population compared to mice studies, in addition to the difficulties to obtained samples and to follow subject over years, both prevent clear identification of the mechanisms and biological processes involved.

### Maternal influence on offspring’s microbiome shapes lasting health consequences

With the mother playing a major role in shaping the offspring’s microbiome, as discussed above, the lasting impact of such perturbations warrants further attention. While these microbiome disruptions may be relatively minor compared to the effects of germ-free conditions or antibiotic treatment—both commonly used in mouse studies to model early-life microbiome perturbation—they can still have significant consequences. Maternal dietary fiber intake has been reported in multiple studies to influence offspring health. A low-fiber diet has been shown to promote offspring susceptibility to intestinal inflammation [23], diet-induced obesity [23], and severe lower respiratory infections [166], while maternal supplementation with β-glucan has demonstrated beneficial effects on offspring cognitive development [167]. In a human study involving 639 mother–infant pairs with a family history of allergic disease, Pretorius et al. reported an association between higher maternal intake of resistant starch and a reduced incidence of infant wheeze, after controlling for a range of environmental and demographic risk factors [168]. Increased maternal fat intake has been implicated in driving disease susceptibility in the next generation. Indeed, maternal high-fat diet has been linked to increased susceptibility to sepsis, food allergies, and autoimmune conditions [169], as well as intestinal inflammation [170–172] and neurological defect [173, 174]. In a US-based cohort of 349 mother–infant dyads, analysis leveraging multiple linear regression models adjusted for potential confounders revealed that maternal fat intake was associated with increased infant body fat percentage at 6 months of age [175]. However, this association was not observed in the Nurture study, an observational birth cohort from the Southeastern U.S. investigating the relationship between maternal diet and infant adiposity [176]. Such discrepancy highlights the challenges of assessing such associations, given the multiple factors influencing infant microbiome and health in the human population. These findings hence warrant further investigation, especially in humans, as fiber reduction and increased fat intake are central features of modern Western diet. Moreover, food additive consumption, another hallmark of modern dietary patterns, has been reported to have lasting consequences on offspring health. Maternal consumption of non-nutritive sweeteners has been associated with metabolic dysregulation in offspring [177], while dietary emulsifiers consumption has been linked to transgenerational disease susceptibility, including negative effects on neuropsychological health [178], low-grade intestinal inflammation, and increased susceptibility to DSS-induced colitis and diet-induced obesity [22]. However, mechanisms by which maternal diet influences the offspring’s microbiome and subsequent health remain poorly understood. In the study by Zou et al. antibiotic treatment in offspring from low-fiber-fed dams was sufficient to prevent increased susceptibility to diet-induced obesity, suggesting that the effects of maternal diet are mediated through lasting microbiome alterations [23]. In recent studies, including ours, a cross-fostering approach was sufficient to either prevent or transfer the long-term susceptibility to disease, indicating that *in utero* modulation by maternal diet may not be involved [22, 71]. These heterogeneous findings highlight the complexity of understanding how maternal diet impacts offspring microbiome and health. Multiple mechanisms, potentially synergistic or diet-specific, may be at play, including (i) direct *in utero* effects of the maternal diet, (ii) *in utero* influences of the maternal microbiome, (iii) postnatal transmission of maternal microbiome and modulation via breast milk, (iv) behavioral changes, and (v) offspring’s immune responsiveness to its microbiome. Given the widespread dietary shifts in modern societies, alongside with rising incidence in non-communicable diseases with a chronic inflammatory component, understanding these mechanisms responsible for maternal influences appears crucial to develop strategies to promote long-term health across generations.

## Discussion

The early-life microbiome plays a pivotal role in shaping immune development and long-term health, with maternal influences serving as critical determinants of offspring microbiome composition and function. Perturbations in maternal microbiome due to dietary alterations can lead to lasting consequences, increasing offspring susceptibility to metabolic, inflammatory, and immune-related diseases. While associations between the maternal environment—including modulation of the microbiome—and offspring’s microbiome development and health have been extensively studied, causal relationships and underlying mechanisms remain poorly understood and warrant further investigation. Moreover, recent studies highlight the individualized nature of microbiome responses to a given diet [179–181], with such inter-individual variation adding complexity to the transgenerational impact of maternal diet on the offspring’s microbiome and health. One emerging area of interest is the additional contribution of paternal microbiome to offspring health. Although maternal transmission remains dominant, recent studies suggest that paternal factors, including diet, may influence early-life microbial colonization and immune programming [78, 182–184]. Investigating the interplay between paternal and maternal microbiome could provide a more comprehensive understanding of early-life microbial inheritance and its functional consequences. Another key aspect requiring further exploration is the functional role played by the early-life microbiome beyond its taxonomical composition. While shifts in microbial communities have been linked to disease susceptibility, we, among others, reported that functional aspects, such as the pro-inflammatory potential of the early-life microbiome, are associated with poor health outcome [22, 185], while SCFA have been reported as crucial for T_REG_ expansion [17]. Defining the specific impact of bacterial metabolism, mucin-degrading activity, and pro-inflammatory molecule synthesis in early life remains a challenge. However, addressing this will enhance our mechanistic understanding of host- microbiome interactions during this critical period.

Given the profound impact of early-life microbiome, targeted modulation strategies have gained interest as potential therapeutic interventions. Pre- and probiotic supplementation, as well as dietary intervention in mothers during the pregnancy and lactation periods, are promising avenues to impact neonatal microbiota composition in a beneficial manner [186–190]. Indeed, a study involving supplementation of gestating dams with *B*. *longum* reported that vertical transmission of this bacterium provided offspring with protection against inflammation susceptibility [186]. Similarly, supplementation with *L. rhamnosus* in rats was sufficient to confer beneficial metabolic effects on the offspring, protecting them from the adverse consequences of a maternal obesogenic diet [187]. In humans, maternal intake of *B*. *lacti*s and *L*. *rhamnosus* has been shown to modulate TLR expression in the placenta [188], while study investigating maternal prebiotic supplementation reported significant differences in infant microbiota at one year of age [189]. However, the long-term effects on infant health—particularly in relation to allergy—remain to be assessed in future follow-up [189]. Results from these pilot studies nonetheless suggest that the efficacy of probiotic interventions may be strain-specific and context-dependent, altogether pointing toward the need for further research into strain selection, timing, different populations as well as long-term impacts. In addition, an innovative approach to modulate infant microbiome involves the use of maternal factors or modifying infant formula composition to better mimic the microbial and metabolic cues provided by maternal milk. This approach was investigated by Holst et al. who performed a clinical study on infant formula supplemented with five different HMOs [191]. Such intervention resulted in a shift of infant microbiome both at the composition and functional level closer to that of breastfed infants, suggesting beneficial effects, also the lasting impact remains to be investigated. An other approach involves the supplementation of formula milk with probiotic, which have been reported to efficiently modulate microbiome’ composition and function in infant receiving such formula [192–194]. Altogether, these approaches appear promising for infant’s microbiome modulation, but the long-term beneficial effects on health, as well as the precise identification of microbial species and functions involved in early-life mechanisms that promote lasting health, require further characterization to refine such preventive strategies.

To conclude, the accumulating evidences discussed above underscore the profound influence played by the maternal environment on shaping an infant’s microbiome and subsequent health. While further studies are needed to develop therapeutic strategies leveraging this route for microbiome modulation, a deeper mechanistic understanding of transgenerational microbiome perturbations in early life remains essential to elucidate their contribution to disease etiology. The widespread use of food additives, increased fat and reduced fiber content in modern diets may contribute to transgenerational shifts in microbiome composition and function, potentially influencing offspring health and playing a role in the rise of non-communicable inflammatory diseases observed over recent decades. Notably, as major dietary shifts toward modern diet emerged in the 1970 s [195, 196], the current generation is experiencing (i) direct exposure to modern diets and their associated adverse health effects, cumulated with (ii) potential transgenerational influences stemming from parental consumption of such diets. Distinct phenotypes have already been reported between the first generation exposed and the second generation, which experiences both direct exposure and potential transgenerational effects in a cumulative manner [197]. This importantly suggests that such interactions may significantly shape the health status of the present generation. Therefore, understanding these environmental pressures appears crucial for developing public health strategies aimed at preserving beneficial microbial ecosystems during early life and mitigating the long-term health consequences of modern dietary patterns.

## Data Availability

No datasets were generated or analysed during the current study.

## Abbreviations

NCD: Non-communicable diseases
IBD: Inflammatory bowel disease
SCFA: Short-chain fatty acid
TEDDY: The Environmental Determinants of Diabetes in the Young
PRR: Pathogen recognition receptors
TLR: Toll-like receptor
IRAK: IL-1 receptor-associated kinase
NKT: Natural killer T
GAP: Goblet cell associated antigen passage
Ach: Acetylcholine
EGFR: Epidermal growth factor receptor
HMOs: Human milk oligosaccharides
IgA: Immunoglobulin A
AhR: Aryl hydrocarbon receptor
